# Mr. Ross, on the Gibraltar Fever

**Published:** 1805-09-01

**Authors:** James M'Grigor

**Affiliations:** London


### To Dr. BATTY.

Sir,

THE enclosed extract of a letter from Thomas Ross, Esq. Surgeon to the Forces at Gibraltar, gives some further account of the fever which raged so fatally there. I only regret that the account is so short from Mr. Ross, who is so well qualified to have given a longer and more complete one. Mr. Ross, from twenty years experience of fever in the East and West Indies, China, Egypt, and Gibraltar, could have given some valuable remarks and comparison of fever as appearing in these various regions.

Your's, respectfully,

*London, Aug.* 10, 1805. JAMES M'GRIGOR.

---

### Symptoms of the Fever which prevailed at Gibraltar in September, October, November, and December, 1804.

It commences with the usual symptoms of fever; sometimes vomiting with the cold fit, but not always; when the hot fit comes on there is a violent pain in the head, chiefly across the forehead, throbbings of the temporal and carotid arteries; the eyes inflamed and watery; from the distension of their small vessels with blood, they have the sensation of being swollen, and give pain when moved; there is violent pain in the loins and calves of the legs; the face is flushed; the skin hot and dry, and when felt leaves a burning sensation at the fingers-end for several seconds; there is great prostration of strength, and generally anxiety and depression of spirits; but in some instances there is the greatest unconcern and indifference as to their situation, from which circumstance the patients were frequently lost, by not reporting themselves in time to be benefited by medicine. The tongue in general was white and moist, sometimes perfectly clean, sometimes the centre was white, the point and sides clean, red, and shining; the thirst in some cases was very great, while some had no thirst; the urine was high coloured; the bowels in general costive, but when they were not so, or when purgatives were given, the stools were uncommonly fetid; little reliance could be put on the pulse as to strength or quickness; in some it exceeded 130 in a minute, and in others it did not exceed 60 during the whole course of the disease. The eye was by much the best index to be guided by.

These

These were the symptoms of the first stage of the disease, which, if not relieved by Nature (which was very seldom the case) or by medicine, in twenty-four hours, the eyes became more suffused and dull, the heat of the skin continued, and all the other symptoms increased till about the end of the second day, when a dull heavy pain was felt at the pit of the stomach, which was soon followed by an effort to vomit; the dullness of the eyes continuing to increase and assuming a dirty yellow glassy appearance, which peculiar appearance was always a fatal symptom. The patient now becomes very restless and delirious; the skin puts on a slight yellow tinge, beginning about the neck, and which in a few hours changes to a dull yellow livid colour; the irritability of the stomach continues, and every thing is rejected; the vomiting becomes constant; what is brought up is a dirty brownish coloured liquor like the washings of Port wine bottles; it gets darker and thicker, and has now more the appearance of coffee-grounds; the desire for cold water now comes on, the restlessness increases, and frequent attempts are made to get out of bed; the delirium is generally expressed in mutterings, but sometimes the convulsions are violent and the ravings frantick; the urine is secreted in small quantity, and sometimes all secretion is suspended; invariably a fatal symptom, and which in some instances occurs early in the fever. Cases have occurred in which not a drop of urine has been passed during the whole course of the disease, yet these patients rarely complained of any inconvenience in consequence. Petechiæ now appear about the breast and arm-pits, and then spread over the whole body; the vomiting continues, attended with hiccup, and a cold, greasy, clammy moisture on the skin, of a peculiar and cadaverous smell; the yellow colour of the countenance acquires a darker hue, and it is strongly marked by the expression of horror; sometimes there are tremors and convulsive motions of the eyes and muscles of the face or limbs; constant muttering; partial intervals of recollection, during which the patient seems sensible of his state, and approaching dissolution, although unable to express himself in words; the extremities are cold; the pulse sinks; and on the third, fifth, or seventh day he dies; in some instances the whole train of symptoms were run through and terminated by death in thirty-six hours from the first attack.

This disease frequently ran quickly on to the putrescent state, when the tongue was covered with a dark brown crust crust; no thirst; early delirum; the skin soft, of a dirty yellow colour, and in point of temperature below the natural standard; profuse cold clammy sweats; very fœtid stools; tension of the abdomen; hæmorrhages of a very dark colour from the nose, mouth, stomach, and anus; livid petechial eruption, black vomiting, hiccup, coma, death.

Sometimes the black vomiting and all the other symptoms subsided, the secretions and pulse appeared natural, the stomach became perfectly retentive both of medicines and nourishment, the senses perfectly collected, the spirits revived, and the patient appeared confident of recovery, and continued so for one or even two days; and when there was every reason to think all danger past, he suddenly, and when least expected, sunk, and expired without a groan or struggle. There is one thing to be observed, that when these illusive appearances took place, the eye still retained its inflamed and glassy appearance.

